# Ferroptosis as a target mechanism in heart and kidney disease

**DOI:** 10.1172/JCI202823

**Published:** 2026-09-15

**Authors:** Simar J. Singh, Baljash Cheema, Hossein Ardehali

**Affiliations:** 1 Department of Medicine and Sarver Heart Center, University of Arizona College of Medicine – Tucson, Tucson, Arizona, USA.; 2 Department of Medicine, Northwestern University School of Medicine, Chicago, Illinois, USA.

## Abstract

Ferroptosis is a distinct form of regulated cell death driven by lipid peroxidation and redox imbalance. Since its formal recognition in 2012, ferroptosis has emerged as a central pathway linking metabolic stress and oxidative injury to both physiologic and pathologic processes. Its functions extend from tissue sculpting during embryogenesis and tumor suppression to pathologic contributions in neurodegeneration, cardiovascular disease, liver and kidney injury, cancer, and inflammatory disorders. Despite these advances in our understanding of ferroptosis, critical questions remain regarding its precise regulation, context-specific consequences, and interactions with other cell death pathways. Continued progress in identifying biomarkers, defining context-specific roles, and developing selective modulators will be essential to translate ferroptosis biology into clinical therapies with broad impact. Here, we describe the current state of our understanding of the role of ferroptosis in physiology and its potential as a target mechanism in heart and kidney disease.

## Introduction

Regulated cell death (RCD) is a critical biological process that maintains tissue homeostasis, drives development, and eliminates damaged or harmful cells. In contrast with accidental cell death, which occurs as an immediate and uncontrolled response to extreme cellular damage, RCD proceeds through genetically encoded signaling pathways that can be modulated by pharmacological or genetic interventions, even when triggered by injurious stimuli.

While RCD was once synonymous with apoptosis, numerous distinct forms of RCD have now been identified, each with unique molecular and morphological characteristics (1). Ferroptosis, a term first coined in 2012 by Dixon et al., is a form of RCD mediated by lipid peroxidation (2). Unlike apoptosis or necroptosis, ferroptosis occurs independently of caspase activation, autophagy machinery, or necrosome components and cyclophilin D, and is driven by the uncontrolled generation of reactive oxygen species (ROS) and lipid peroxides causing membrane damage (2–4). Ferroptotic cells exhibit distinct morphological features, including shrunken mitochondria with dense membranes and preserved nuclei (5, 6).

The concept of a unique, iron-regulated form of RCD predates the coining of the word *ferroptosis*. In the 1950s, Harry Eagle’s studies on cystine deprivation identified a unique cell death pattern linked to glutathione (GSH) depletion that could be inhibited by iron chelators or antioxidants (7–10), features later recognized as hallmarks of ferroptosis (11–15). In the following decades, it was recognized that lipid peroxidation and eventual cell death from this process can be initiated through nonenzymatic and enzymatic mechanisms (3, 16).

Seminal work by Ursini and colleagues in the 1980s identified phospholipid hydroperoxide glutathione peroxidase (now called GPX4) as a selenium-dependent enzyme that protects membranes from lipid peroxidation, laying the groundwork for its later link to ferroptosis (17, 18). Genetic studies later confirmed that loss of *Gpx4* resulted in embryonic lethality in mice, highlighting its critical role in preventing oxidative damage (19–21). In the early 2000s, the identification of compounds such as erastin and RAS-selective lethal small molecule 3 (RSL3), which induce cell death by inhibiting cystine uptake and disrupting redox balance, further supported the notion of a peroxidation-based, nonapoptotic form of RCD (2, 22). The discovery of ferrostatin-1 (FER-1), an inhibitor of ferroptosis, cemented ferroptosis as a distinct RCD mechanism (23). These findings, spanning redox biology and metabolism, led to the formal definition of ferroptosis in 2012. Since then, a growing body of evidence has highlighted the diverse physiologic and pathologic roles of ferroptosis, underscoring its potential as a therapeutic target.

## Mechanisms of ferroptosis

### Lipid peroxidation.

**Figure 1:**
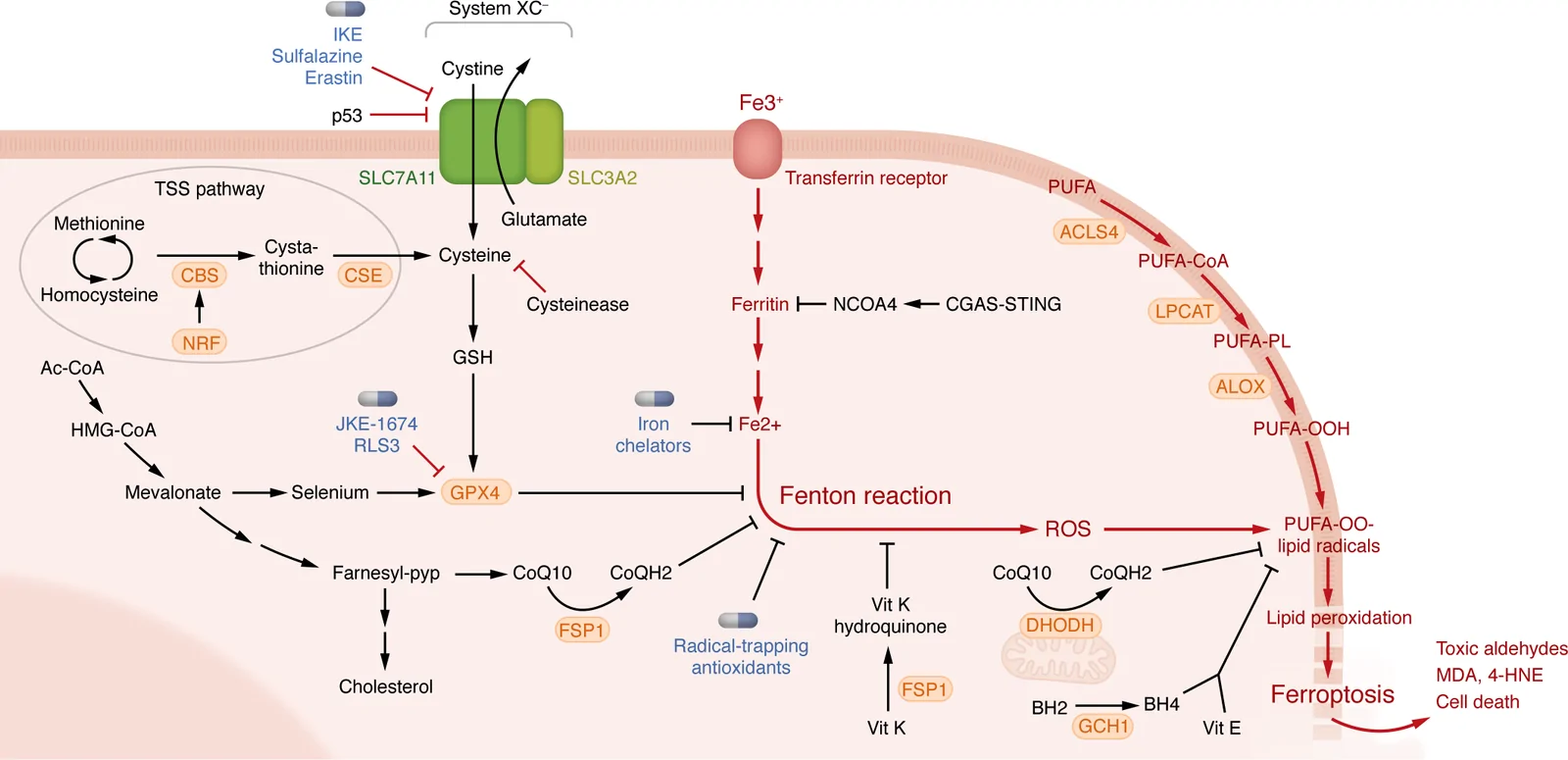
Ferroptosis propagation and inhibition. Ferroptosis is a form of regulated cell death caused by overwhelming lipid peroxidation. The Fenton reaction converts cytosolic hydrogen peroxide or membrane-residing lipid hydroperoxide (L-OOH) into highly reactive hydroxyl radicals, which initiate lipid peroxidation. Alternatively, PUFAs incorporated into the membrane undergo oxidation, resulting in the formation of L-OOH. Multiple mechanisms modulate or inhibit ferroptosis. System Xc^−^ imports cystine into the cell, which is reduced to cysteine, the rate-limiting precursor for GSH synthesis. Alternatively, methionine may be used to generate cysteine via the methionine cycle and TSS pathway. GPX4 uses GSH to reduce L-OOH to nontoxic lipid alcohols, thereby protecting cellular membranes from oxidative damage. Other antioxidant defense systems, such as the FSP1-CoQ10 system and BH4/BH2, independently suppress ROS accumulation and inhibit ferroptosis. IKE, imidazole ketone erastin; PUFA-PL, polyunsaturated fatty acid phospholipid; TSS, transulfuration; FSP1, ferroptosis suppressor protein 1; BH4/BH2, tetrahydrobiopterin/dihydrobiopterin; HMG-CoA; 3-hydroxy-3-methylglutaryl-coenzyme A; Ac-CoA; acetyl CoA.

Ferroptosis is mechanistically characterized by overwhelming lipid peroxidation, a process driven by both enzymatic and nonenzymatic pathways (Figure 1). In addition to the Fenton reaction, which generates reactive hydroxy radicals from iron and hydrogen peroxide, lipid peroxidation can be initiated by ROS such as alkoxyl (RO•) and peroxyl (ROO•) radicals, which arise from metal-catalyzed decomposition of lipid hydroperoxides and other cellular processes, including mitochondrial respiration, xenobiotic redox cycling, and inflammatory oxidative bursts in the presence of catalytic iron or copper (24). PUFAs, such as arachidonic acid (AA), are particularly vulnerable to oxidation due to their methylene double bonds, making them key targets in ferroptosis. Enzymes like acyl-CoA synthetase long-chain family member 4 (ACSL4) and lysophosphatidylcholine acyltransferase 3 (LPCAT3) facilitate the incorporation of PUFAs into membrane phospholipids. Once incorporated into the membrane, these PUFAs undergo oxidation, primarily catalyzed by lipoxygenases, cyclooxygenases, and cytochrome P450 oxidoreductases, resulting in the formation of L-OOH (25, 26). Subsequently, L-OOH can generate highly reactive lipid radicals, which further propagate peroxidation and ferroptosis (27).

As lipid peroxidation progresses, biophysical changes occur, including increased membrane stiffness and tension (28). This, combined with the activation of mechanosensitive ion channels like Piezo1, causes ion imbalance and osmotic swelling of the cell (4). The culmination of these processes is the release of toxic lipid by-products, such as malondialdehyde (MDA) and 4-hydroxynonenal (4-HNE), and ultimately cell rupture (27, 29). These by-products further damage cellular components, including proteins and DNA, thereby exacerbating cellular injury and amplifying the pathological consequences of ferroptosis.

### GSH-GPX4 axis and antioxidant defense.

The GSH-GPX4 axis is the central defense mechanism against ferroptosis, preventing the accumulation of lipid peroxides that lead to cell death (6, 21). As a selenoprotein, GPX4 uses reduced GSH to reduce L-OOH to nontoxic lipid alcohols (L-OH), thereby protecting cellular membranes from oxidative damage (30). As noted above, the importance of GPX4 is highlighted by genetic studies showing that in mice, *Gpx4* knockout results in embryonic lethality and conditional GPX4 deletion leads to spontaneous ferroptosis in tissues like the kidney (6, 19, 20). GPX4 exists in 3 isoforms, cytosolic (cGPX4), mitochondrial (mGPX4), and nuclear (nGPX4), with cGPX4 being the most crucial for preventing ferroptosis in most tissues (31–33).

The cystine/glutamate antiporter (System Xc^−^), composed of solute carrier family 7 member 11 (SLC7A11 or xCT) and solute carrier family 3 member 2 (SLC3A2), also plays a critical role in GSH synthesis and ferroptosis activity (34, 35). This antiporter imports cystine into cells in exchange for glutamate. Once inside the cell, cystine is reduced to cysteine, the rate-limiting precursor for GSH synthesis. By providing the cell with cystine, System Xc^−^ is essential for GSH production, and its inhibition by compounds like erastin or sulfasalazine leads to GSH depletion, subsequent GPX4 inactivation, and the triggering of ferroptosis (22, 36).

### Other key regulators and pathways.

In addition to GPX4, other antioxidant defense systems suppress ferroptosis (Table 1). FSP1, also known as AIFM2, operates independently of GPX4 by reducing ubiquinone (CoQ10) and vitamin K to their hydroquinone forms, which act as lipophilic radical-trapping antioxidants, and in the case of vitamin K underlies its antidotal role against warfarin-induced anticoagulation (37, 38). This reduced form directly counteracts lipid peroxidation at the plasma membrane. The FSP1-CoQ10 system provides critical protection when the GPX4-GSH axis is compromised, and this redundancy emphasizes the evolutionary pressure to protect cells from lipid peroxidation through multiple mechanisms (39). FSP1 localizes to the plasma membrane via myristoylation and confers ferroptosis resistance in part by mitigating lipid peroxidation, including in cancer cells (39). Inhibition of FSP1 sensitizes cells to ferroptosis, particularly when combined with other pro-ferroptotic stimuli (37, 39).

As described above, among the most critical enzymatic regulators of ferroptosis are ACSL4 and LPCAT3, which drive the incorporation of PUFAs into cellular membranes (25). ACSL4 catalyzes the production of polyunsaturated fatty acyl-coenzyme A (PUFA-CoA) from AA, which is then esterified into phosphatidylethanolamine (PE) by LPCAT3 (40–42). These lipid species are particularly susceptible to oxidation, making their incorporation into membranes a key event in ferroptosis execution. The activity of ACSL4 is crucial for ferroptosis sensitivity, as its ablation confers protection against ferroptotic cell death (25, 43, 44). Moreover, ACSL4 activity is regulated by a feed-forward loop involving protein kinase CβII (PKCβII), which senses initial lipid peroxidation and activates ACSL4, further promoting PUFA incorporation (45). In contrast, ACSL3 preferentially activates monounsaturated fatty acids (MUFAs), which are less susceptible to peroxidation and thus confer protection against ferroptosis (46, 47).

The tumor suppressor p53 also plays a pivotal, context-dependent role in ferroptosis. p53 can downregulate SLC7A11, leading to GSH depletion, increased lipid peroxidation, and susceptibility to ferroptosis (48, 49). Interestingly, mutant forms of p53 (such as p53-3KR) retain the ability to promote ferroptosis despite being defective in their traditional functions, such as cell cycle arrest and apoptosis (48). In contrast, under certain conditions, p53 can upregulate antioxidant defense genes, including GPX4, and inhibit dipeptidyl peptidase-4 (DPP4) activity, thus suppressing ROS accumulation and preventing ferroptosis (50, 51).

Nuclear factor erythroid 2-related factor 2 (NRF2), a transcription factor, plays a key role in ferroptosis suppression by upregulating ferroptosis-related proteins, including components of System Xc^−^ (52, 53), and proteins involved in iron sequestration and release, such as ferritin and heme oxygenase-1 (HO-1) (54–62). NRF2 activation enhances cellular resistance to ferroptosis by promoting these cytoprotective mechanisms (63–65). However, high NRF2 expression can sometimes enhance ferroptosis sensitivity in cancer cells by upregulating multidrug resistance proteins that export GSH (66), highlighting the context-dependent nature of NRF2’s role in ferroptosis.

Other modulators further fine-tune ferroptosis sensitivity by reinforcing antioxidant defenses or amplifying lipid peroxidation. GTP cyclohydrolase-1 (GCH1) drives synthesis of BH4/BH2, which function as potent radical-trapping antioxidants. Beyond directly scavenging lipid peroxyl radicals, BH4/BH2 remodel phospholipid composition by preserving species with polyunsaturated fatty acyl tails, and promote endogenous CoQ10 synthesis, together establishing a GCH1–BH4–phospholipid axis that confers ferroptosis resistance independently of the canonical GPX4/GSH system (67). Complementing this, BH4 cooperates with vitamin E (α-tocopherol) to synergistically prevent lipid autoxidation, and its regeneration via dihydrofolate reductase sustains this protective capacity (68).

Within mitochondria, dihydroorotate dehydrogenase (DHODH) has been proposed to operate parallel to mGPX4 by reducing CoQ10 to CoQH2, thereby suppressing lipid peroxidation (69), though the relative contribution of the mGPX4 isoform remains a subject of debate. Pharmacological inhibition of DHODH selectively triggers ferroptosis in GPX4-low tumors and synergizes with ferroptosis inducers in GPX4-high tumors (69). However, recent evidence suggests that these effects may be partially attributed to off-target inhibition of FSP1 rather than DHODH activity alone (70).

Arachidonic (C20:4) and adrenic (C22:4) acid–containing PEs are critical substrates for ferroptosis, generating hydroperoxy-phosphatidylethanolamines (Hp-PEs) detoxified by GPX4. PLA2G6 (iPLA2β) mitigates ferroptosis by metabolizing Hp-PEs, protecting trophoblasts from hypoxia/reoxygenation injury, whereas Lp-PLA2 (PLA2G7) promotes resistance by regulating intracellular PE/lyso-PE balance, with its inhibition by darapladib synergizing with GPX4 inhibitors to suppress tumor growth (71, 72). Thus, phospholipases are context-dependent checkpoints of ferroptosis and potential therapeutic targets.

An additional regulator is the transsulfuration (TSS) pathway, which provides an alternative source of cysteine when extracellular cystine is limited or under oxidative stress, with cystathionine β-synthase (CBS) and cystathionine γ-lyase catalyzing the key rate-limiting steps. Notably, CBS upregulation by NRF2, SP1/3, or nuclear factor Y (NFY) enhances redox homeostasis and confers resistance to ferroptosis (73–76).

## Physiological roles of ferroptosis

Although first described in pathological contexts, ferroptosis is now recognized as an evolutionarily conserved physiological process with essential roles in development, tissue remodeling, immune regulation, and metabolic homeostasis. During embryogenesis, ferroptosis functions as a sculpting mechanism. In avian limb morphogenesis, ferroptotic waves propagate across millimeter-length fields, shaping tissue architecture in a spatiotemporally coordinated manner (77). In mice, *Gpx4* deficiency causes midgestational lethality and markedly increases sensitivity to oxidative stress and irradiation (19, 20). In human embryonic stem cells, iron accumulation and GPX4 depletion drive dissociation-induced ferroptosis, which is suppressed by ferrostatin-1 or iron chelation, establishing ferroptosis as a regulator of stem cell viability (78). Additionally, GPX4 loss in hematopoietic stem cells causes reduced protein synthesis, defective reticulocyte maturation, and ineffective erythropoiesis, thereby conferring a selective vulnerability to ferroptosis (79). These findings suggest that ferroptosis is tightly regulated during development to regulate cell number and tissue patterning.

Ferroptosis also contributes to tissue differentiation and homeostasis in adult organisms. In the epidermis, the epigenetic regulator mixed-lineage leukemia 4 (MLL4) promotes differentiation in part through transcriptional control of ferroptosis-related lipoxygenases (*Alox12*, *Alox12b*, *Aloxe3*), linking ferroptotic pathways to skin barrier formation and tumor suppression (80). Similarly, age-related ferroptotic activity increases across multiple tissues, including kidney, liver, spleen, ovary, and bone marrow, and does so in association with iron accumulation, suggesting that ferroptosis is a conserved physiological mechanism of tissue turnover that becomes maladaptive with aging (81). In *C*. *elegans*, iron buildup and GSH depletion during late life prime tissues for ferroptosis, with inhibition of lipid peroxidation in this setting extending life span and health span, suggesting ferroptosis is a contributor to healthy aging in model organisms (82).

The immune system also utilizes ferroptosis both as a regulator of immune cell fitness and as an effector mechanism against target cells. CD8^+^ T cells activated by immunotherapy induce ferroptosis in tumor cells through IFN-γ–dependent downregulation of SLC7A11 and SLC3A2, thereby restricting cystine uptake and driving lipid peroxidation (83). IFN signaling also enhances ACSL4-mediated incorporation of AA into phospholipids, sensitizing tumor cells to ferroptosis and amplifying T cell–mediated cytotoxicity (44). Conversely, immune cell subsets themselves require protection from ferroptosis. Follicular helper T cells rely on the selenium-GPX4 axis for survival, with loss of GPX4 triggering ferroptosis and impairing germinal center formation and antibody responses (84). Tumor-infiltrating CD8^+^ T cells may also undergo ferroptosis when CD36-mediated fatty acid uptake drives lipid peroxidation, leading to functional exhaustion; blocking CD36 or inhibiting ferroptosis restores their cytotoxicity and improves checkpoint blockade efficacy (85).

Ferroptosis also intersects with redox balance. By selectively eliminating cells with toxic levels of ROS, ferroptosis maintains tissue integrity. In cancer, this vulnerability can be therapeutically exploited: PUFAs, particularly under conditions of tumor acidosis, induce ferroptosis and inhibit tumor growth, an effect enhanced by diglyceride acyltransferase inhibitors that prevent lipid droplet sequestration (86). Likewise, lung adenocarcinomas select for high expression of the iron–sulfur cluster biosynthetic enzyme NFS1 to resist oxidative stress and ferroptosis in the high-oxygen pulmonary environment, illustrating how ferroptotic pressure shapes tumor evolution (87).

Ferroptosis is integrally linked to tumor suppression. For example, TP53 enhances ferroptotic sensitivity by repressing SLC7A11 and regulating metabolic genes involved in redox control (48, 49, 88). However, p53 also exhibits antiferroptotic functions, including induction of iPLA2β, activation of the p21-glutathione axis, and inhibition of DPP4, leading to ferroptosis resistance and revealing a dual and highly context-specific role in ferroptosis regulation (50, 51, 89). Additional tumor suppressors converge on ferroptotic pathways, including BAP1 and AMER1, which promote ferroptosis through repression or degradation of SLC7A11, thereby sensitizing cancer cells to lipid peroxidation (90, 91). Similarly, KEAP1 and ARF suppress NRF2-driven antioxidant programs, removing a major barrier to ferroptosis, while noncanonical suppressors such as MLL4 regulate the balance of ferroptosis-promoting lipoxygenases (ALOX12/12B/12E3) and inhibitory factors such as GPX4 and SLC7A11 (63, 80, 92, 93).

Taken together, these studies establish ferroptosis as a physiological mechanism for developmental tissue sculpting, homeostatic cell turnover, immune regulation, and tumor suppression. While indispensable in these contexts, its dysregulation underlies diverse pathological states ranging from cancer and metabolic disease to neurodegeneration and inflammatory disorders (Table 2). Below, we focus on heart and kidney disease, 2 areas where abundant experimental evidence supports important roles of ferroptosis in pathology. However, we acknowledge that cancer, aging, and neurodegeneration represent rapidly expanding domains of ferroptosis research that cannot be comprehensively addressed in a single review.

## Ferroptosis in cardiovascular disease

Ferroptosis has emerged as a critical player in the pathogenesis and progression of cardiovascular disease (CVD), with its involvement suggesting a fundamental role in cardiac cell death and tissue remodeling. Given the heart’s high metabolic rate and iron demands, cardiomyocytes are particularly vulnerable to ferroptotic injury, especially during pathological conditions such as ischemia, inflammation, and oxidative stress.

### Myocardial ischemia/reperfusion injury and myocardial infarction.

Ferroptosis is thought to play an important role in myocardial ischemia/reperfusion injury (MIRI) and myocardial infarction (MI) (94, 95). Reperfusion paradoxically exacerbates myocardial injury and cardiomyocyte death due to the overproduction of ROS and lipid peroxidation (96). The restoration of blood flow generates a burst of ROS, which overwhelms cellular antioxidant defenses, including the GSH-GPX4 pathway, thereby promoting ferroptosis (95, 97).

In a coronary ligation model, it was found that apoptosis and necrosis were responsible for early MIRI, while ferroptosis was prevalent during prolonged reperfusion (95, 98). Moreover, during reperfusion, FER-1 delivery provided greater protection against MIRI injury than inhibitors of other cell death pathways (95). Reperfusion leads to progressive increases in ACSL4, iron, and MDA, which occur alongside GPX4 loss; concurrently, the iron chelator deferoxamine reduces reperfusion injury, indicating that ferroptosis is a reperfusion-specific, rather than ischemic, event (98).

The lipoxygenase ALOX15 and its metabolite 15-hydroperoxyeicosatetraenoic acid were shown to drive cardiomyocyte ferroptosis in reperfusion via Pgc1α degradation, while both ML351, a selective inhibitor of ALOX15, and cardiomyocyte-specific deletion of ALOX15 protected myocardium against reperfusion injury and preserved left ventricular (LV) function (95). Additionally, ALOX12 was shown to drive cardiomyocyte damage through 12-hydroxyeicosatetraenoic acid accumulation and suppression of AMPK signaling. Given its role in PUFA peroxidation, ALOX12 likely amplifies ferroptotic injury, with genetic or pharmacologic inhibition protecting myocardium following reperfusion injury across species (99).

Recent work has demonstrated that during MIRI, double-stranded DNA (dsDNA)–cyclic GMP-AMP synthase (cGAS)–stimulator of interferon genes (STING) promotes lipid peroxidation and ferroptosis via degradation of GPX4 (100). In STING-deficient mice, MIRI failed to induce the expected decrease in ACSL4 or the degradation of GPX4, indicating a key upstream role for STING in regulating ferroptotic cell death (100). Additionally, overexpression of GPX4 in STING-activated mice was sufficient to abrogate MIRI-induced LV dysfunction, highlighting a clear role for GPX and ferroptosis in the pathogenesis of MIRI (100).

Endoplasmic reticulum stress (ERS) promotes apoptosis via the p53-upregulated modulator of apoptosis protein and C/EBP homologous protein (CHOP) (101). Ferroptosis inducers activate the PERK/eIF2α/ATF4/CHOP pathway to trigger ERS (102), while inhibition of System Xc^–^ exacerbates oxidative stress and ERS (34). Additionally, ROS and phospholipid oxidation products generated during ferroptosis further stimulate ERS (103, 104). In rat models, CHOP-mediated ERS aggravates MIRI, highlighting ferroptosis-induced ERS as a key driver of reperfusion injury (34).

Several reagents have been shown to protect against ferroptotic cell death during MIRI (Table 3). Cyanidin-3-glucoside (C3G), an anthocyanin abundant in red-purple fruits and vegetables, attenuates oxidative stress and suppresses ferroptosis through GPX4 upregulation and modulation of USP19–Beclin1–NCOA4–LC3–dependent ferritinophagy, resulting in reduced infarct size (105). Resveratrol, a polyphenolic compound, protects against MIRI through a nearly identical mechanism (106). Oxidized phosphatidylcholines (OxPCs) accumulate during reperfusion, where they impair mitochondrial energetics, disrupt calcium handling, suppress GPX4, and promote ferroptotic cardiomyocyte death. Both FER-1 and the OxPC-neutralizing antibody E06 mitigate this injury, highlighting OxPCs as potential therapeutic targets in MIRI (107). Etomidate protects against MIRI by preserving mitochondrial function and suppressing ferroptosis in a dose-dependent pattern, effects that were abolished by the ferroptosis inducer erastin (108). Flavonoids such as naringenin, icariin, and hederagenin protect against MIRI by attenuating oxidative stress and ferroptosis through distinct mechanisms, including activation of the Nrf2/System Xc^–^/GPX4 axis (naringenin), suppression of ERS- and IRE1/JNK–driven mitochondrial dysfunction (icariin), and inhibition of ALOX5-dependent lipid peroxidation (hederagenin) (109–111).

### SIC.

SIC is a frequent but reversible cause of acute ventricular dysfunction in critically ill patients, characterized by systolic or diastolic impairment independent of coronary artery disease (112). Ferroptosis plays a central role in SIC pathogenesis, which is driven by oxidative stress, GSH depletion, and GPX4 downregulation, promoting lipid peroxidation and cardiomyocyte death. Iron overload via ferritinophagy and lipocalin 2–mediated dysregulation of iron metabolism further amplifies ferroptotic injury (113).

Multiple regulators for SIC have been identified. ICA69 promotes ferroptosis through STING-driven lipid peroxidation, while its deficiency alleviates cardiomyocyte damage in sepsis models (114). TMEM43 protects against LPS-induced cardiac dysfunction by maintaining GPX4/SLC7A11 and suppressing p53-driven ferroptosis (115). Similarly, TRPM7 upregulation exacerbates ferroptosis via ERS, while knockdown attenuates myocardial injury. Circadian disruption in SIC, particularly downregulation of Bmal1, sensitizes cardiomyocytes to ferroptosis through the AKT/p53 pathway (116).

Therapeutic interventions targeting ferroptosis in SIC show promise (Table 3). Dexmedetomidine attenuates septic myocardial injury by restoring GPX4, suppressing HO-1–mediated iron release, and reducing lipid peroxidation (117). Natural compounds such as puerarin (via AMPK/GPX4 signaling) (118), matrine (via PI3K/AKT) (119), tectorigenin (via Smad3 suppression) (120), and resveratrol (via Sirt1/Nrf2 and miR-149/HMGB1 pathways) (121, 122) inhibit ferroptosis and ameliorate SIC. Additional strategies include nicorandil, which modulates TLR4/SLC7A11 signaling (123); platelet-rich plasma, which activates AKT/mTOR signaling (124); and nanozyme-based therapies that scavenge ROS, inhibit ferroptosis, and reduce inflammation in SIC (124).

### Atherosclerosis.

Atherosclerosis is a chronic inflammatory disease of the arterial wall and a leading cause of MI, stroke, and peripheral vascular disease (125). Ferroptosis has emerged as a driver of atherosclerosis, linking lipid peroxidation to endothelial dysfunction. Iron accumulation in vascular macrophages promotes Fenton reactions, generating ROS and lipid peroxides that accelerate foam cell formation and vascular injury (126). Elevated expression of ACSL4 and prostaglandin-endoperoxide synthase in advanced plaques marks ferroptosis activation, while heme oxygenase-1 further exacerbates lipid peroxidation and ferroptotic damage (127). Clinical evidence supports this association, as plasma lipid peroxides are elevated in patients with coronary and peripheral artery disease and correlate with disease severity (128).

Mechanistic studies highlight multiple regulators of ferroptosis in atherosclerosis. GPX4 protects endothelial cells and macrophages from oxidized lipid toxicity, and its overexpression delays lesion progression (129, 130). Conversely, myeloid GPX4 deficiency enhances foam cell formation and accelerates atherogenesis (131). Mitochondrial metabolism is also implicated. For example, deficiency of prenyl (decaprenyl) diphosphate synthase, subunit 2, which supports CoQ10 biosynthesis, promotes ferroptosis in endothelial cells, while its restoration activates NRF2 and protects against atherosclerosis (132). Similarly, sirtuin 1 (SIRT1) activation inhibits ferroptosis and inflammation in foam cells, underscoring the link between ferroptosis and redox and autophagy pathways (126).

Multiple other modulators of ferroptosis in atherosclerosis have been identified. The long noncoding RNA lnc-MRGPRF-6:1 promotes ox-LDL–induced macrophage ferroptosis by suppressing GPX4 and is elevated in patients with coronary artery disease (133). Cigarette tar, through NF-κB–induced hepcidin/FPN/SLC7A11 dysregulation, drives macrophage ferroptosis and accelerates plaque progression (134). Additionally, radiation-induced atherosclerosis is mediated by endothelial ferritinophagy/ferroptosis via the p38/NCOA4 pathway (135).

Importantly, ferroptosis is not only a pathogenic driver of atherosclerosis but also a therapeutic target (Table 3). Treatment with the ferroptosis inhibitor FER-1 reduces lesion size, lipid peroxidation, and endothelial dysfunction in ApoE^−/−^ mice (127). Iron chelators, such as deferoxamine and desferricoprogen, reduce plaque burden by attenuating oxidative stress and lipid peroxidation, foam cell formation, and endothelial activation (136). Natural compounds also show efficacy, with micheliolide suppressing macrophage ferroptosis by activating NRF2 signaling (137), icariin reducing endothelial ferroptosis through transcription factor EB-mediated autophagy (138), and hydroxysafflor yellow A preventing diabetic atherosclerosis by modulating the miR-429/SLC7A11 axis (139). Estrogen has also been shown to protect against postmenopausal atherosclerosis by inhibiting endothelial ferroptosis via NRF2/GPX4 signaling (140).

### HF.

HF, a leading cause of morbidity and mortality worldwide, affects over 6.7 million Americans, with prevalence projected to exceed 11 million by 2050 (141). Ferroptosis has emerged as a contributor to cardiomyocyte loss and ventricular dysfunction in HF. Hallmarks of ferroptosis, including lipid peroxidation, are consistently observed in HF models (142, 143).

Mechanistically, cardiomyocyte ferroptosis is driven by disruption of antioxidant defenses, and loss of ferritin heavy chain or ferritin H impairs iron buffering, leading to lipid peroxidation and contractile dysfunction, which can be rescued by ferroptosis inhibitors such as FER-1 (143, 144). Similarly, deficiency of GPX4 exacerbates doxorubicin-induced cardiomyopathy (DIC), whereas GPX4 overexpression or ferroptosis blockade is cardioprotective (143, 145). Expression of the transcription factor BTB and CNC homology 1, by repressing antioxidant and iron-regulatory genes, enhances susceptibility to ferroptosis and worsens myocardial injury (146). In contrast, regulators such as phosphoglycerate mutase family member 5, SIRT1, and mTOR protect against ferroptotic death by stabilizing antioxidant signaling pathways, including NRF2/SLC7A11/GPX4 (147–149).

Therapeutic studies further underscore ferroptosis as a tractable target in HF (Table 3). Natural compounds including resveratrol (via SIRT1/p53/SLC7A11), stachydrine, puerarin, and astragaloside IV restore GPX4 function and limit ferroptotic remodeling (142, 147, 150, 151). Standard therapies may also exert antiferroptotic effects: statins suppress ferroptosis (152), and both atorvastatin and the ROCK inhibitor fasudil enhance NRF2 signaling and redox balance (153). Importantly, ferroptosis inhibition has shown efficacy in mitigating DIC, radiation-induced cardiomyopathy, and alcoholic cardiomyopathy (95, 154, 155).

In summary, ferroptosis is a critical player in both acute and chronic cardiovascular conditions. Targeting ferroptosis through inhibitors of lipid peroxidation, iron chelation, and modulation of key regulatory pathways holds promise for improving outcomes in CVDs.

## Ferroptosis in kidney disease

Ferroptosis has been shown to contribute to renal damage in various types of kidney injury, including ischemia/reperfusion injury (IRI), rhabdomyolysis, and drug-induced nephrotoxicity, as well as in the chronic progression of diseases like diabetic nephropathy and polycystic kidney disease.

### Acute kidney injury.

Ferroptosis has emerged as a central mechanism driving acute kidney injury (AKI), with IRI representing one of the best-characterized contexts. During reperfusion, excess ROS initiates lipid peroxidation, promoting tubular necrosis. Genetic ablation studies confirm ferroptosis as the dominant form of RCD in the kidney. Inducible deletion of GPX4 precipitates acute renal failure and death in mice, directly linking the GSH-GPX4 axis to renal ferroptosis (6). Intravital microscopy studies further demonstrated that ferroptosis, rather than necroptosis, is the primary mode of tubular death in IRI (156, 157). The heightened susceptibility of proximal tubular cells to IRI reflects downregulation of GPX4 and SLC7A11, coupled with increased ACSL4, which amplifies lipid peroxidation.

Ferroptosis is also implicated in rhabdomyolysis-induced AKI, in which muscle-derived myoglobin releases free iron upon degradation, catalyzing lipid peroxidation and ferroptosis in renal tubules. FER-1 and related analogs mitigate this process (158). In exertional heatstroke, elevated serum myoglobin strongly predicts AKI risk and is mechanistically linked to ERS-induced ferroptosis (159).

Nephrotoxicity induced by the chemotherapeutics cisplatin and adriamycin also converges on ferroptotic pathways through GSH depletion and ROS generation. Recent drug-repurposing screens identified multiple agents approved by the US FDA (i.e., carvedilol, rifampicin, and omeprazole) with antiferroptotic activity via lipid peroxyl radical scavenging, which reduced cisplatin-induced tubular injury (160). Such findings suggest immediate translational potential for mitigating drug-induced nephrotoxicity.

Beyond acute cell death, ferroptosis contributes to maladaptive repair and fibrosis in AKI. Single-cell transcriptomics revealed that severe ischemic injury drives proximal tubule cells into a persistent pro-inflammatory state characterized by GSH depletion and ferroptotic stress, which in turn sustains inflammatory signaling and fibrosis (161). Complementary single-cell analyses demonstrated that maladaptive, profibrotic tubule clusters upregulate cytokines and myeloid chemotactic factors, with druggability screens identifying pyroptosis and ferroptosis as critical pathways whose inhibition shifts repair toward adaptive regeneration (162). Thus, ferroptosis not only executes acute necrosis but also orchestrates the trajectory of renal repair.

Pharmacologic inhibition of ferroptosis provides robust protection in multiple AKI models (Table 4). FER-1 preserves renal function in folic acid–induced AKI, attenuating lipid peroxidation and inflammation (163). FER-1 also prevents tubular necrosis in rhabdomyolysis-induced AKI by scavenging lipid peroxides, though without altering mitochondrial ROS or lysosomal injury (158). Liproxstatin-1 rescues GPX4-knockout mice from ferroptosis-driven renal failure (6). More recently, next-generation ferrostatins, such as SRS16-86 and UAMC-3203, have shown greater stability and in vivo potency, outperforming FER-1 in models of IRI and inducible renal tubular ferroptosis (156, 164).

Endogenous and dietary metabolites also modulate ferroptotic susceptibility. Vitamin K functions as a potent radical-trapping antioxidant through FSP1-mediated reduction to hydroquinones, suppressing lipid peroxidation and protecting against IRI-induced renal damage (38, 165). Similarly, 7-dehydrocholesterol, a cholesterol biosynthetic intermediate, has been identified as a natural antiferroptotic metabolite that shields membranes from peroxidation, and pharmacologic manipulation of this pathway alleviates renal IRI (166). The MUFA oleic acid also mitigates iron overload toxicity and tubular ferroptosis by remodeling lipid composition, reducing polyunsaturated phospholipid substrates (161). In rhabdomyolysis-induced AKI, natural compounds, such as curcumin, provide renoprotection by scavenging ROS, activating HO-1, and suppressing TLR4/NF-κB signaling (167).

### CKD.

Ferroptosis also plays a pivotal role in the progression of CKD, where persistent lipid peroxidation promotes tubular injury, inflammation, and fibrosis. In diabetic nephropathy, ferroptotic stress is evident in both tubular epithelial and mesangial compartments. High glucose and TGF-β downregulate SLC7A11 and GPX4, reducing cystine uptake and GSH synthesis, thereby priming renal cells for lipid peroxidation and ferroptosis. High-mobility group box 1 protein (HMGB1) signaling further amplifies this process by activating the TLR4/NF-κB axis and suppressing NRF2-dependent antioxidant pathways, leading to accumulation of ROS, MDA, and other ferroptotic markers (168, 169). Inhibition of ferroptosis with FER-1 or HMGB1 knockdown restores redox balance, attenuates inflammation, and mitigates renal injury, highlighting ferroptosis as a critical mediator of diabetic kidney damage.

ADPKD represents another setting in which ferroptosis drives disease progression. In mice, loss of *Pkd1* disrupts metabolic homeostasis by reducing expression of cystine transporters and ferroportin, thereby lowering GSH and GPX4 activity, while simultaneously increasing expression of iron importers such as transferrin receptor 1 (TfR1) and divalent metal transporter 1, along with HO-1 (170). This ferroptosis-prone metabolic state sensitizes renal epithelial cells to lipid peroxidation. The accumulation of toxic lipid peroxidation products, including 4-HNE, paradoxically promotes proliferation of surviving cystic cells through AKT, STAT3, S6, and Rb activation, thereby accelerating cyst expansion. Inhibition of ferroptosis with FER-1 delays cyst growth in *Pkd1*-mutant models, identifying ferroptosis as a novel therapeutic target in ADPKD (170).

In hypertensive nephropathy, chronic high blood pressure induces mitochondrial dysfunction, with HO-1 upregulation serving as both a marker and amplifier of ferroptotic stress (171, 172). These changes contribute to tubular injury and interstitial fibrosis, but inhibition of ferroptosis with ACSL4 knockdown or FER-1 administration has been shown to attenuate renal damage in models of hypertensive nephropathy (172–174). Similarly, in IgA nephropathy (IgAN), renal biopsies reveal increased iron deposition and elevated TfR1 expression in mesangial cells, correlating with cell proliferation and disease progression (175). Iron chelators and ferroptosis inhibitors ameliorate renal injury in experimental IgAN, suggesting that ferroptosis directly contributes to glomerular pathology (175).

Membranous nephropathy also exhibits a ferroptotic signature. Experimental models of Heymann nephritis demonstrate reduced GPX4 and increased ACSL4 and TfR1 expression, coupled with activation of ferritinophagy via microtubule-associated protein 1 light chain 3 and nuclear receptor coactivator 4, which mobilizes iron stores and exacerbates lipid peroxidation. These molecular changes parallel the development of proteinuria, hypoproteinemia, and glomerular damage, underscoring ferroptosis as a mechanistic driver of membranous nephropathy progression (176).

## Conclusions and future directions

Ferroptosis has rapidly emerged as a central mechanism linking lipid peroxidation and cellular redox homeostasis to diverse pathological states, ranging from degenerative disorders to cancer. Considerable progress has been made in defining its biochemical hallmarks, including the essential roles of GPX4, System Xc^–^, FSP1, and ACSL4, as well as the broader metabolic networks that regulate susceptibility. Yet, critical challenges remain before ferroptosis-targeted therapies can be translated into clinical practice.

First, there is a vital need for robust biomarkers to detect ferroptosis in vivo. While lipid peroxidation products, GPX4 levels, and ACSL4 expression provide indirect evidence, they lack specificity and dynamic resolution. The development of imaging probes, ferroptosis-specific biosensors, and validated pharmacodynamic markers will be essential to guide early-phase clinical trials and patient stratification.

On the therapeutic front, drug development faces several obstacles. Current ferroptosis inhibitors and inducers largely target master regulators such as GPX4 or act as radical-trapping antioxidants, raising concerns about systemic toxicity and off-target effects. Importantly, the intrinsic susceptibility of healthy stem cell populations to ferroptosis raises a critical translational concern, as ferroptosis-inducing therapeutics may compromise tissue regeneration and long-term homeostasis by inadvertently targeting normal stem cell compartments (78, 79). A shift toward modulating accessory regulatory complexes or combining ferroptosis-based therapies with established modalities (i.e., immune checkpoint blockade, chimeric antigen receptor T cell therapy, chemotherapy, or targeted kinase inhibition) may allow safer and more durable outcomes.

Importantly, recent clinical trials underscore the complexity of translating ferroptosis-targeted therapies into human disease. In the FAIRPARK-II randomized trial, the iron chelator deferiprone failed to slow disease progression and was associated with worse clinical outcomes in patients with early-stage Parkinson’s disease (177). Similarly, in Alzheimer’s disease, despite compelling preclinical evidence linking iron accumulation and lipid peroxidation to neurodegeneration, the 3D trial demonstrated that deferiprone did not meet primary cognitive endpoints and was associated with significant adverse effects (178). These findings highlight that ferroptosis is not simply a uniform pathogenic mechanism, but rather a tightly context-dependent process in which the timing of intervention, disease stage, and specificity of iron-targeting strategies are likely critical determinants of therapeutic success.

At the same time, the clinical translation of ferroptosis modulators has evolved substantially from early experimental compounds toward more sophisticated therapeutic candidates with improved pharmacokinetic and pharmacodynamic properties. Early ferroptosis inducers, such as the GPX4 inhibitor RSL3, provided critical mechanistic insight but were limited by poor bioavailability and unfavorable pharmacokinetics that restricted their utility to preclinical studies (179). In contrast, RLS-1496 recently became the first selective GPX4 modulator to enter human clinical trials, demonstrating safety and successful target engagement in phase I studies for inflammatory skin diseases as of late 2025 (NCT07340697). Similar progress has occurred in targeting the FSP1/CoQ10 pathway. While the original FSP1 inhibitor exhibited substantial off-target effects and limited in vivo applicability, the next-generation compound icFSP1 employs a phase-separation mechanism to induce potent antitumor activity in vivo (180). These advances, together with the June 2025 approval of ifupinostat for lymphoma and the ongoing phase III evaluation of vatiquinone (NCT04577352), suggest that the field is transitioning from broadly acting redox-active compounds toward precision ferroptosis modulators capable of overcoming many of the physiological and pharmacologic barriers that previously hindered clinical development.

Additionally, the immunological consequences of ferroptosis remain incompletely understood. Ferroptotic tumor cells can release immunosuppressive lipid mediators, potentially dampening antitumor immunity, while certain immune cells, such as CD8^+^ T cells, are themselves vulnerable to ferroptotic death (83). Achieving cell-specific precision targeting will therefore be crucial to maximize efficacy while preserving host immunity.

From a translational perspective, identifying patient populations most likely to benefit from ferroptosis therapies should be a priority. Tumor genotype, metabolic dependencies, and microenvironmental cues including hypoxia and microbiota shape ferroptotic sensitivity. Integrating ferroptosis biology with genomic and metabolomic profiling could enable predictive algorithms to select responsive subgroups. Moreover, insights from nonmalignant conditions such as neurodegeneration, IRI, and infection may uncover convergent ferroptotic mechanisms and therapeutic vulnerabilities relevant to multiple clinical scenarios.

In conclusion, ferroptosis occupies a unique position at the intersection of metabolism, redox regulation, and immunity, making it both a compelling biological process and a therapeutic opportunity. Its successful translation into the clinic will depend on resolving disease-specific contexts, establishing robust biomarkers, and developing precision strategies to manipulate ferroptosis safely. As these challenges are addressed, ferroptosis has the potential to shift from a mechanistic insight to a transformative approach for treating a diverse array of human diseases.

## Conflict of interest

HA serves as an expert witness for health-related legal cases.

## Funding support

This work is the result of NIH funding, in whole or in part, and is subject to the NIH Public Access Policy. Through acceptance of this federal funding, the NIH has been given a right to make the work publicly available in PubMed Central.

- NIH grants R01HL180711, 1R01HL175366, R61HL179722 (to HA).
- Leducq Foundation grant (to HA).

## Figures and Tables

**Table 3:**
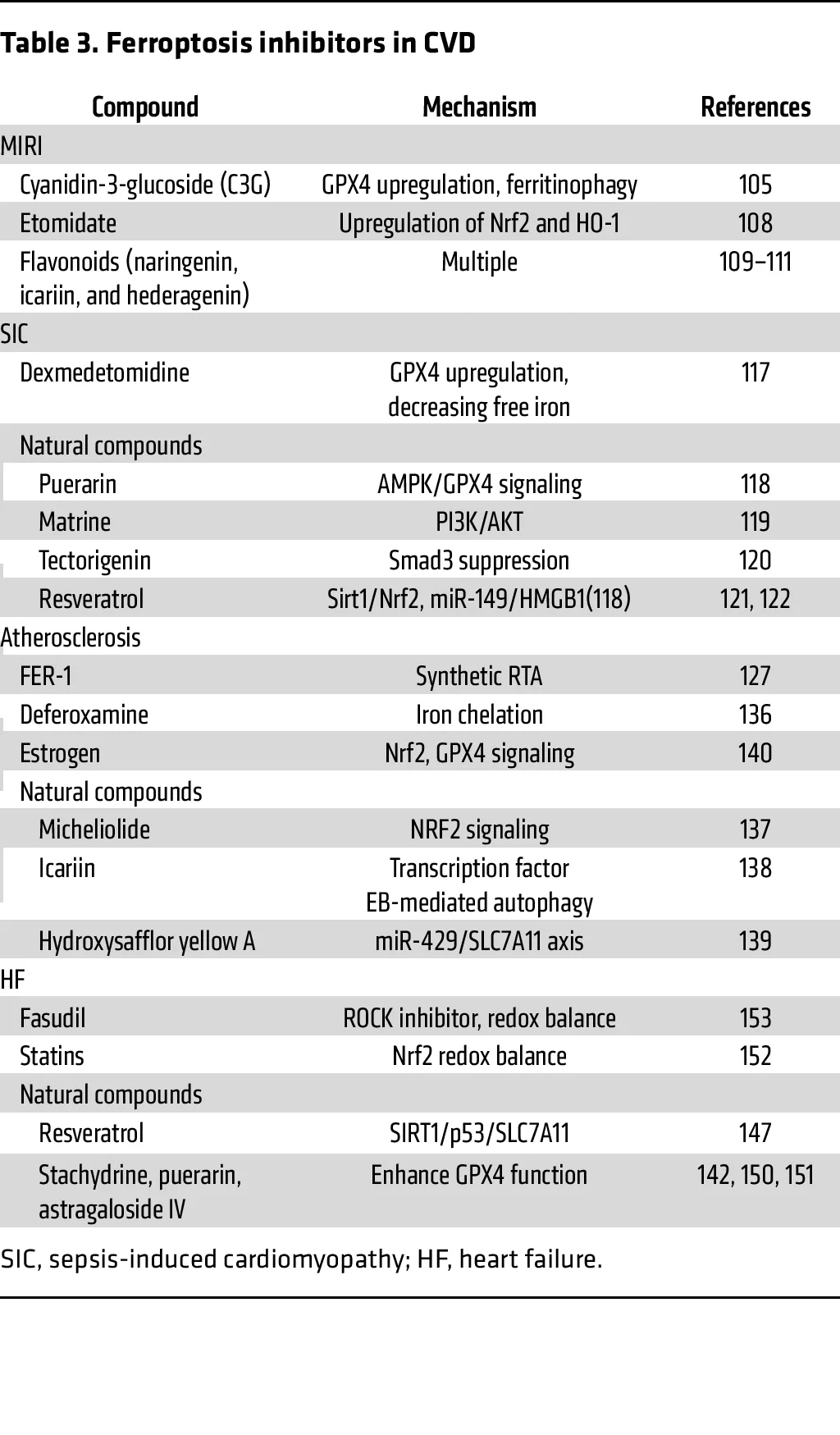
Ferroptosis inhibitors in CVD

**Table 2:**
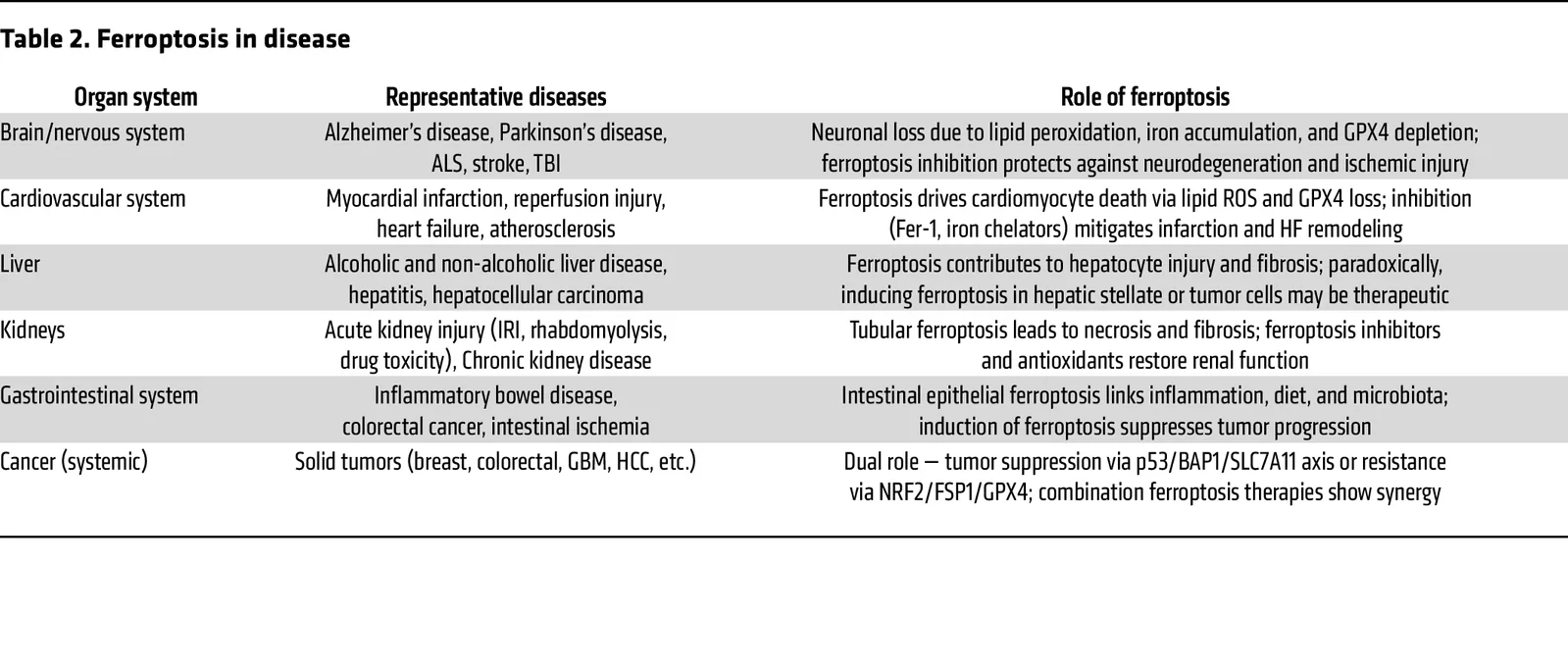
Ferroptosis in disease

**Table 1:**
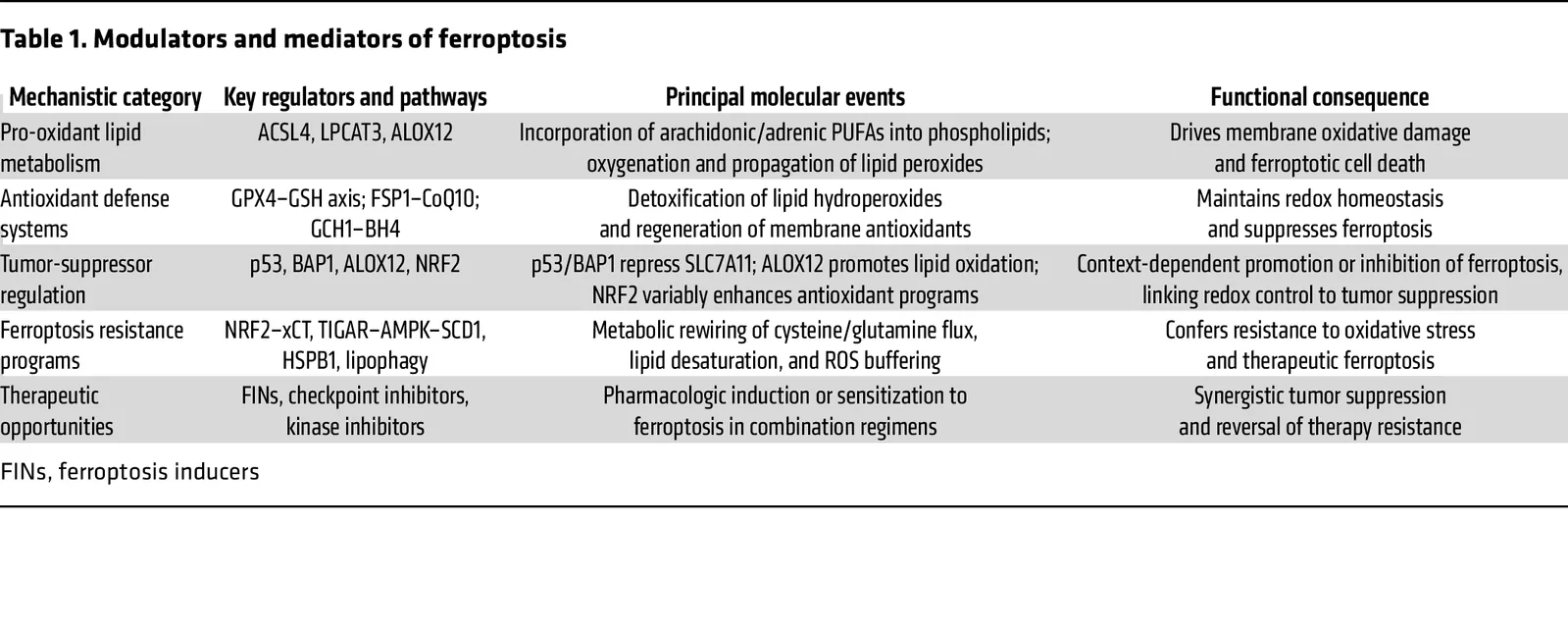
Modulators and mediators of ferroptosis

**Table 4:**
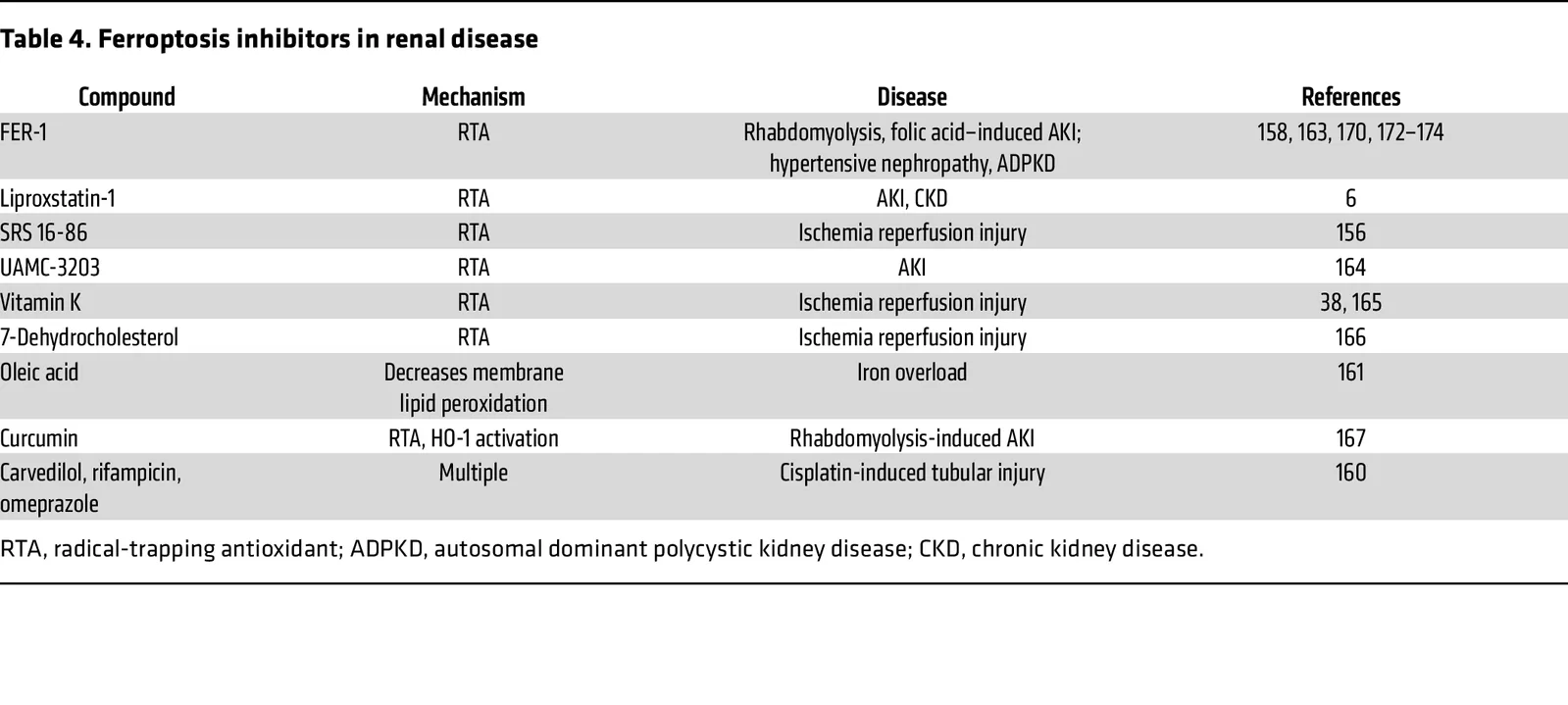
Ferroptosis inhibitors in renal disease

## Version 1 09/15/2026

Electronic publication
